# Role of bronchoalveolar lavage in the diagnosis of acute exacerbations of idiopathic pulmonary fibrosis: a retrospective study

**DOI:** 10.1186/s12890-015-0066-3

**Published:** 2015-07-10

**Authors:** Frunze Petrosyan, Daniel A. Culver, Anita J. Reddy

**Affiliations:** Hospital Medicine Department, Johnston Health, University of North Carolina Health System, 509 N Brightleaf Blvd, Smithfield, NC 27527 USA; Respiratory Institute, Cleveland Clinic, 9500 Euclid Avenue, Cleveland, OH 44195 USA

**Keywords:** Idiopathic pulmonary fibrosis, Interstitial lung disease, Bronchoalveolar lavage, Acute respiratory failure

## Abstract

**Background:**

It has been recognized that despite previous stability some patients with idiopathic pulmonary fibrosis (IPF) experience acute clinical deteriorations called acute exacerbations of idiopathic pulmonary fibrosis (AEX-IPF). We hypothesized that pulmonary infection can be excluded based on clinical and laboratory data and that bronchoscopy with BAL is not mandatory in the diagnostic work-up of suspected AEX-IPF.

**Methods:**

In this retrospective study we identified patients with acute respiratory failure who were evaluated for AEX-IPF at the Cleveland Clinic between January 2002 and December 2011. Univariate and multivariate analysis were performed with predefined risk factors and final diagnosis of AEX-IPF and pulmonary infection. All tests were performed at a significance level of 0.05.

**Results:**

A total of 77 patients met the study inclusion criteria. Of these patients 47 (61 %) were diagnosed with AEX-IPF. Bronchoscopy was more likely to be performed in patients who were on cytotoxic medications (*p* < 0.05). In most cases the diagnosis of AEX-IPF versus pulmonary infection was based on combination of other microbiological, clinical, radiologic data and clinical judgment. A total of 10 patients out of 14 (71 %) with a final diagnosis of pulmonary infection were on steroids on admission versus 21 out of 63 patients (33 %) with other final diagnosis (*p* = 0.024, OR 7.817, 95 % CI 1.31–46.64).

**Conclusions:**

Exclusion of infection in our IPF patient cohort was mostly based on factors other than diagnostic bronchoscopy with BAL. Based on our results we suggested an algorithm for management of IPF patients presenting with acute respiratory failure.

## Background

Idiopathic pulmonary fibrosis (IPF) is the most common form of idiopathic interstitial pneumonia. It has been recognized that some patients with IPF experience acute clinical deteriorations, despite previous stability. Most of these deteriorations are idiopathic; others are secondary to infection, left heart failure, pulmonary embolism, pneumothorax and other identifiable causes of acute lung injury. These episodes of idiopathic acute deteriorations have been termed acute exacerbations of IPF (AEX-IPF). Diagnostic consensus criteria for AEX-IPF were suggested by Collard et al. in 2007 [1] and include: previous or concurrent diagnosis of IPF, unexplained worsening or development of dyspnea within the past 30 days, specific high resolution chest computed tomography (CT) pattern and no evidence of infection in the absence of alternative causes that are specifically mentioned in the consensus statement.

According to these criteria, AEX-IPF can only be diagnosed if there is no evidence of pulmonary infection by endotracheal aspirate or bronchoalveolar lavage (BAL). Evaluation of samples should include studies for routine bacterial organisms, opportunistic pathogens such as pneumocystis jiroveci (PJP), and common viral pathogens including influenza A and B, parainfluenza 1–4, respiratory syncytial virus A and B, human metapneumovirus, adenovirus and coronaviruses. Those patients who have no endotracheal aspirate or BAL available are classified as having “suspected acute exacerbation of IPF”.

A study by Wootton et al. [2] did not detect viral infection in most cases of AEX-IPF. In this study four of 43 BAL samples from AEX-IPF patients were positive for respiratory viruses and 15 for non-respiratory viruses compared to no viral detection in stable IPF controls. This study suggested that isolation of these viruses has no proven clinical significance, so BAL viral studies might not be helpful in management of these patients [2]. AEX-IPF cases occur more commonly in winter and spring, suggesting that some of them might have unidentified infections etiology, even despite extensive microbiological workup [3]. On the other hand, some patients with suspected AEX-IPF have microbiological evidence of infection but also have clinical and imaging characteristics of AEX-IPF [4]. Completing the course of broad spectrum antibiotics might be reasonable even if there is a low suspicion of pulmonary infection and AEX-IPF is the working diagnosis especially if there is clinical improvement. Procalcitonin guided antibiotic use has been tested in various respiratory infections, including IPF, and was shown to reduce the antibiotic exposure in AEX-IPF patients [5]. This strategy is not routinely recommended and should be further explored.

In a recent proposal by Johannson and Collard, authors also question the mandatory role of BAL in the diagnostic workup of AEX-IPF patients, considering poor sensitivity of microbiological tests and the risk of worsening hypoxemia with bronchoscopy in non-intubated patients with baseline high oxygen requirements [6]. Some risk factors favor the diagnosis of AEX-IPF, such as obesity, subacutely worsening dyspnea, decline in forced vital capacity and pulmonary hypertension [7,8]. Identified risk factors should be incorporated into clinical decision tools and treatment algorithms.

In this study, we hypothesize that pulmonary infection can be excluded based on clinical and laboratory data and that bronchoscopy with BAL is not mandatory in the diagnostic work-up of patient with suspected AEX-IPF. We also looked for risk factors and patient characteristics that might help to guide treatment decisions.

## Methods

This retrospective study identified patients with idiopathic pulmonary fibrosis and acute respiratory failure who were evaluated for AEX-IPF at the Cleveland Clinic between January 2002 and December 2011. Study claims compliance with Helsinki Declaration. Cleveland Clinic institutional board review approved the study protocol and determined that it meets criteria for waiver for consent.

### Inclusion criteria

Adult patients with known history of IPF who presented with possible AEX-IPF or new patients that eventually were diagnosed to have AEX-IPF as an initial manifestation of the disease and patients with full predefined information available in the electronic medical records at the Cleveland Clinic for the following diagnoses: idiopathic pulmonary fibrosis and acute exacerbation of idiopathic pulmonary fibrosis.

### Exclusion criteria

Patients with missing information in the medical records, and patients status post lung transplant.

### Statistical methods used

Continuous measures were described as means, standard deviations, and percentiles. Categorical measures were summarized using frequencies and percentiles. The Pearson’s chi-square test or Fisher’s exact test was used to assess the associations between the binary groups and categorical measures. The two sample *T*-test were used to evaluate the relationship between binary groups and continuous measures. All tests were performed at a significance level of 0.05. SAS 9.3 software (SAS Institute, Cary, NC) was used for all analyses.

## Results

A total of 77 patients met the study inclusion criteria, of which 37 were females and 40 were males. Of these patients, 47 (61 %) were diagnosed with AEX-IPF (Table 1). Bronchoscopy with BAL was done in 38 % of all patients (29 procedures), as well as 38 % in the subgroup of patients eventually diagnosed with AEX-IPF (18 procedures). In 6 of these 29 procedures, bronchoscopy was performed prior to administration of antibiotics.

**Table 1:** Table represents final diagnosis in IPF patients admitted with acute respiratory failure. Two most common final diagnosis were AEX-IPF and pulmonary infection

Final diagnosis	
AEX-IPF	47
Pulmonary infection	14
IPF progression	5
Acute CHF	2
NSIP flare	2
Hypoglycemia and respiratory failure	1
COPD exacerbation	1
Pulmonary embolism	1
Transtracheal oxygen catheter related problem	1
Pneumomediastinum	1
Ischemic heart disease	1
Bronchogenic carcinoma	1
Total number of cases	77

Bronchoscopy was more likely to be performed in patients who were on cytotoxic medications, but it did not depend on gender, smoking history, prior steroid therapy or any other patient characteristics (Table 2). Diagnosis of infection was made when BAL, tracheal aspirate, sputum culture or blood culture was found positive and not considered to be a contaminant. Of the 14 patients who were diagnosed with pulmonary infections, two had fever on admission (*p* = 0.15), and 12 had white blood count greater than 11.0 k/ul (*p* = 0.17) and a total of 57 out of 77 patients were started on broad spectrum antibiotics. Six patients had a BAL performed, but with only one identified case of infection. In this one patient, BAL was positive both for PJP and cytomegalovirus and blood culture was positive for vancomycine resistant enterococcus (VRE). An additional patient had a BAL performed which grew methicillin sensitive staphylococcus aureus (MSSA), but the final diagnosis was AEX-IPF. Both of these patients were treated with antibiotics prior to BAL being performed. Tracheal aspirate cultures were done for six patients (four of them had BAL done with no growth), and one patient was positive for influenza A virus. Sputum culture was performed in 26 patients, and two patients grew Stenotrophomonas maltophilia (judged to be contaminant) and Klebsiella pneumoniae respectively. Blood cultures were done in 50 patients, and two patients grew VRE and staphylococcus hominis respectively (latter was judged to be contaminant, Table 3). Of the three patients who had BAL and sputum cultures done at the same time, only one sputum culture was positive for growth (Klebsiella pneumoniae) while the BAL did not show evidence of infection. Mycoplasma IgM, urine streptococcus pneumonia antigen and urine Legionella antigen were checked in six, four, and twelve patients respectively and were negative in all patients.

**Table 2:** Table represents association between IPF patient characteristics on hospital admission and performance of bronchoscopy with BAL. Two patients with missing data on BAL performance were excluded

	Bronchoscopy with BAL done			
Factor	No (*N* = 46)	Yes (*N* = 29)	Total (*N* = 75)	*P*-value
Gender				0.97^a^
Female	22 (47.8 %)	14 (48.3 %)	36 (48.0 %)	
Male	24 (52.2 %)	15 (51.7 %)	39 (52.0 %)	
Tobacco exposure				0.28^a^
Yes	23 (48.9 %)	15 (62.5 %)	37 (53.6 %)	
No	23 (51.1 %)	9 (37.5 %)	32 (46.4 %)	
Prior steroid use				0.85^a^
No	26 (56.5 %)	13 (54.2 %)	39 (55.7 %)	
Yes	20 (43.5 %)	11 (45.8 %)	31 (44.3 %)	
Prior cytotoxic agents				<0.001^b^
No	46 (100.0 %)	17 (73.9 %)	63 (91.3 %)	
Yes	0 (0.0 %)	6 (26.1 %)	6 (8.7 %)	
Antibiotics on admission				0.46^a^
No	35 (76.1 %)	17 (68.0 %)	52 (73.2 %)	
Yes	11 (23.9 %)	8 (32.0 %)	19 (26.8 %)	
Fever on admission				0.99^b^
No	43 (92.5 %)	23 (95.8 %)	60 (93.8 %)	
Yes	3 (7.5 %)	1 (4.2 %)	4 (6.3 %)	
Tachycardia on admission				0.95^a^
No	36 (74.4 %)	18 (75.0 %)	47 (74.6 %)	
Yes	10 (25.6 %)	6 (25.0 %)	16 (25.4 %)	
Tachypnea on admission				0.37^a^
No	16 (23.1 %)	8 (33.3 %)	17 (27.0 %)	
Yes	30 (76.9 %)	16 (66.7 %)	46 (73.0 %)	
ICU care				0.70^a^
No	29 (63.0 %)	17 (58.6 %)	46 (61.3 %)	
Yes	17 (37.0 %)	12 (41.4 %)	29 (38.7 %)	
Procalcitonin				0.60^c^
Mean (SD)	0.4 (0.8)	0.7 (0.6)	0.6 (0.7)	
Range	(0.1–1.6)	(0.1–1.7)	(0.1–1.7)	

^a^Chi-Square

^b^Fisher Exact

^c^ *T*-Test

**Table 3:** Table represents microbiologic data obtained in the study patients with IPF presenting with acute respiratory failure and the positivity rate of the cultures

Type of culture	Total (*N* = 77)	
	No	Yes
Tracheal aspirate obtained	71 (92.1 %)	6 (7.9 %)
Growth on tracheal aspirate	76 (98.7 %)	1 (1.3 %)
Sputum culture obtained	51 (65.3 %)	26 (34.7 %)
Sputum culture positive for infection	75 (97.4 %)	2 (2.6 %)
Blood cultures obtained	27 (34.2 %)	50 (65.8 %)
Blood culture positive	75 (97.4 %)	2 (2.6 %)

Univariate and multivariate analysis were performed with predefined risk factors and final diagnosis of AEX-IPF and pulmonary infection (Tables 4 and 5). Only prior to admission steroid use, which was defined ad daily prednisone intake 10–60 mg, was found to be significantly associated with developing a pulmonary infection, where 10 out of 14 patients (71 %) on steroids were found to have an infection versus only 21 out of 63 patients (33 %) patients who were not on steroids (*p* = 0.024, OR 7.817, 95 % CI 1.31–46.64). Overall mortality in our population cohort was 28.6 %, and this was not significantly different amongst AEX-IPF patients (29.8 %), patients with pulmonary infection (28.5 %) and patients with respiratory failure due to other causes (25 %).

**Table 4:** Multivariable association between final diagnosis of AEX-IPF and patient risk factors. No statistically significant association was revealed

Effect	Odds ratio	95 % CI		*P*-value
Steroids on admission: No vs Yes	2.998	0.881	10.206	0.079
Cytotoxic agents on admission: No vs Yes	1.054	0.156	7.117	0.96
Antibiotics on admission: No vs Yes	1.372	0.352	5.344	0.65
Sputum culture positive: No vs Yes	4.007	0.235	68.279	0.34
Elevated WBC on admission: No vs Yes	1.22	0.37	4.022	0.74
Fever on admission: Yes vs No	1.112	0.086	14.313	0.94
Tachycardia on admission: No vs Yes	1.454	0.396	5.331	0.57
Tachypnea on admission: Yes vs No	1.814	0.472	6.978	0.39

**Table 5:** Multivariable association between final diagnosis of pulmonary infection and patient risk factors

Effect	Odds ratio	95 % CI		*P*-value
Steroids on admission: Yes vs No	7.817	1.31	46.64	0.024^*^
Cytotoxic agents on admission: No vs Yes	2.407	0.196	29.524	0.49
Antibiotics on admission: Yes vs No	2.051	0.308	13.65	0.46
Sputum culture positive: Yes vs No	2.427	0.148	39.718	0.53
Elevated WBC on admission: Yes vs No	1.474	0.268	8.094	0.66
Fever on admission: Yes vs No	1.651	0.109	25.021	0.72
Tachycardia on admission: No vs Yes	1.552	0.201	11.956	0.67
Tachypnea on admission: Yes vs No	1.088	0.142	8.362	0.94

^*^Patients who were on steroids on admission were more likely diagnosed with pulmonary infection then patients who were not on steroids (*p* = 0.024)

## Discussion

Today, BAL technique is standardized [9] and it is often used in the workup of AEX-IPF. Pesci et al. [10] recommended that BAL should be considered in all IPF patients with suspected infection, malignancy or AEX-IPF. Papanikolaou et al. [11], as well as Wuyts et al. [12] state that BAL should be performed if the patient can tolerate the procedure (DLCO > 30 % and P_a_O_2_ > 75 mmHg on supplemental oxygen). The official ATS/ERS/JRS/ALAT statement on pulmonary fibrosis does not give clear recommendations on the diagnostic workup for AEX-IPF [13]. Overall BAL is widely considered a part of the diagnostic workup of a patient with IPF presenting with acute respiratory failure, and is performed for nearly every evaluated patient that can tolerate it, although the predictive usefulness and safety of the procedure has not been fully elucidated.

Several other potentially useful roles of the BAL were recently entertained. It has been shown that BAL samples from some AEX-IPF patients have increased level of pepsin [14] and that treatment with proton pomp inhibitors might have a role in the prevention of exacerbations in these selected patients [15]. This suggests that some IPF exacerbations might be triggered by silent aspiration and those patients do not need treatment with broad spectrum antibiotics. Song et al. showed that measuring percentage of neutrophils in the BAL fluid can be a useful tool to discriminate between pulmonary infection and AEX-IPF but this practice has not been routinely recommended and needs further investigation [16]. If clinical suspicion for drug induced alveolitis or other specific etiology, BAL can be performed tailored to that specific diagnoses, in case the BAL fluid differential count would change the management. For AEX-IPF or infection, no such strong data is available, and one should not base treatment decisions on BAL fluid differential count. In addition, the percentage of neutrophils in the BAL fluid is increased during the AEX-IPF episodes compared to stable patients with IPF and controls, which makes this data less reliable to exclude infectious process [17, 18]. It has also been shown that BAL is not a benign procedure and in fact, is an independent risk factor for IPF exacerbation [19–22]. In a retrospective study it was shown that the risk of AEX-IPF is elevated within 30 days after BAL (RR 4.12; 95 % CI 1.03–12.2), moreover the relative risk of developing AEX-IPF after second or later BAL procedures was estimated to be considerably higher (RR 9.10; 95 % CI 2.27–26.98). In a recent review of the utility of BAL in diffuse parenchymal lung diseases, the role of BAL was critical in the diagnosis of opportunistic infections in patients treated with immunosuppressive therapy [23], but is not necessary in all patients.

In this retrospective study to assess the diagnostic value of bronchoscopy and BAL performed in the work up for suspected AEX-IPF cases we identified patients with a known history of IPF, who presented with acute respiratory failure and were being evaluated for AEX-IPF. AEX-IPF and pulmonary infection were the two most common final diagnoses and a minority of patients were found to have other cardiovascular and pulmonary conditions as a cause of their acute decompensation. The diagnosis of AEX-IPF was not associated with any of the predefined patient characteristics or measurable factors (such as gender, tobacco exposure or vital signs on admission). 38 % of patients had a bronchoscopy with BAL performed as a part of the diagnostic workup and it was more likely to be performed in patients receiving cytotoxic agents. One can only speculate that these patients were considered high risk for pulmonary infection and BAL was done due to high pretest probability. There was no other significant difference between two groups, which allowed further statistical analysis.

It is worth noting that in our cohort only three of 14 patients who had a final diagnosis of pulmonary infection had microbiological confirmation, one each from BAL and blood culture, tracheal aspirate culture and sputum culture. Only prior to admission steroid use was associated with a final diagnosis of pulmonary infection. In most cases, diagnosis of infection was made on the basis of physical examination, clinical history, laboratory/imaging data and clinician judgment. It seems BAL is most helpful when performed in patients with high pretest probability such as patients on steroids or immunosuppressive agents.

Most of our patients were started on broad spectrum antibiotics on admission prior to BAL and completed the course despite negative microbiological workup. We believe this is a common scenario in other centers too and shows that BAL fluid analysis does not change the treatment strategy. In our cohort bronchoscopy with BAL had little influence on the management of the patients which might suggest that patients who present with possible AEP-IPF versus pulmonary infection should be empirically treated with broad spectrum antibiotics and that bronchoscopy with BAL should be performed in selected cases only based on clinical judgment and case scenario, such as current use of steroids or other immunosuppressive agents.

Limitations of our study include relatively small sample size, single center participation and the retrospective nature of the study. Treatment selection biases as well as reliance on expert opinion in many cases for final diagnosis should be considered as well. Cleveland Clinic is a tertiary care center and a patient selection bias also could not be excluded. Our practice is not to use immunosuppressants for maintenance treatment of IFP patients, so our findings may not be translatable in other institutions who have not adopted this practice. Most of our patients were started on antibiotics before BAL could be performed and our conclusions may not be generalized to patient in whom BAL with fluid differential and cultures are done first.

**Fig. 1:**
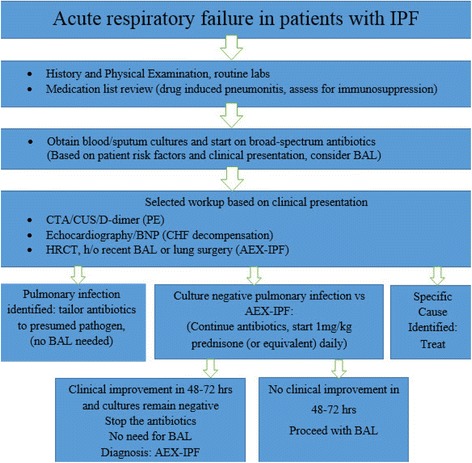
Suggested algorithm for the management of patients with IPF who present with acute respiratory failure. IPF-idiopathic pulmonary fibrosis, BAL-bronchoalveolar lavage, CTA-computer tomographic angiography, CUS-compression ultrasonography, PE-pulmonary embolism, BNP-brain natriuretic peptide, CHF-congested heart failure, AEX-IPF-acute exacerbation of idiopathic pulmonary fibrosis

Therefore, based on our findings in this study, we propose the following algorithm in the management of IPF patients presenting with acute respiratory failure (Fig. 1). IPF patients presenting with acute respiratory failure should first be evaluated for identifiable causes for their deterioration including but not limited to pulmonary infection, congestive heart failure decompensation, aspiration, pulmonary embolism, drug induced complications, and AEX-IPF based on clinical presentation. We suggest that all patients should be initiated on broad spectrum antibiotics upon presentation (including coverage for PJP if clinically indicated), ideally after blood, sputum and, in select cases, BAL cultures are obtained. One should not wait for culture results to initiate antibacterial therapy, but it should be used for de-escalation strategy. If BAL cultures are routinely obtained after initiation of antibiotic therapy, false negative results are likely and make further decisions for de-escalation a guess. Accordingly, we do not suggest BAL for all patients. If BAL can be safely obtained before the antibiotics are given, the diagnostic workup might be different and not reflected by our algorithm as most of our patients did get antibiotics before the BAL. If a non-infectious cause is identified, such as pulmonary embolism or pneumothorax, then antibiotics can be safely discontinued. Bronchoscopy with BAL should be performed in immunocompromised patients on steroids and other cytotoxic drugs, but also for selected patients with worsening respiratory failure despite broad spectrum antibiotics and inconclusive or unrevealing workup. In a retrospective study by Song et al., BAL and/or endotracheal aspiration were performed in 52.8 % of 461 patients highlighting the fact that in real life scenarios BAL is not performed for various reasons despite the universal recommendation and that our algorithm will be suitable for these cases [16]. It is based on small sample size, retrospective data and single center experience and should be used with these limitations in mind.

## Conclusions

Our data support that the decision regarding performance of BAL should be used in conjunction with other historical and clinical data, and in select cases clinician should be able to forego bronchoscopy. Exclusion of infection in our IPF patient cohort was mostly based on factors other than diagnostic bronchoscopy with BAL. Prior to admission steroid use was associated with a final diagnosis of pulmonary infection. Based on our results we suggested an algorithm for management of IPF patients presenting with acute respiratory failure.

## Abbreviations

IPF: Idiopathic pulmonary fibrosis
AEX-IPF: Acute exacerbations of idiopathic pulmonary fibrosis
CT: Computed tomography
BAL: Bronchoalveolar lavage
PJP: Pneumocystis jiroveci
VRE: Vancomycine resistant enterococcus
MSSA: Methicillin sensitive staphylococcus aureus
CHF: Congested heart failure
COPD: Chronic obstructive pulmonary disease
NSIP: Nonspecific interstitial pneumonia
WBC: White blood cell
CTA: Computer tomographic angiography
CUS: Compression ultrasonography
PE: Pulmonary embolism
BNP: Brain natriuretic peptide
